# Coordination of precision grip in 2–6 years-old children with autism spectrum disorders compared to children developing typically and children with developmental disabilities

**DOI:** 10.3389/fnint.2012.00122

**Published:** 2012-12-31

**Authors:** Fabian J. David, Grace T. Baranek, Chris Wiesen, Adrienne F. Miao, Deborah E. Thorpe

**Affiliations:** ^1^Department of Kinesiology and Nutrition, Motor Control and Movement Disorders Group, University of Illinois at ChicagoChicago, IL, USA; ^2^Division of Occupational Science, Department of Allied Health Sciences, The University of North Carolina at Chapel HillChapel Hill, NC, USA; ^3^The Odum Institute, The University of North Carolina at Chapel HillChapel Hill, NC, USA; ^4^Division of Physical Therapy, Department of Allied Health Sciences, The University of North Carolina at Chapel HillChapel Hill, NC, USA

**Keywords:** autism spectrum disorders, developmental delay, motor deficits, motor coordination, temporal motor coordination, precision grip, grip force, load force

## Abstract

Impaired motor coordination is prevalent in children with Autism Spectrum Disorders (ASD) and affects adaptive skills. Little is known about the development of motor patterns in young children with ASD between 2 and 6 years of age. The purpose of the current study was threefold: (1) to describe developmental correlates of motor coordination in children with ASD, (2) to identify the extent to which motor coordination deficits are unique to ASD by using a control group of children with other developmental disabilities (DD), and (3) to determine the association between motor coordination variables and functional fine motor skills. Twenty-four children with ASD were compared to 30 children with typical development (TD) and 11 children with DD. A precision grip task was used to quantify and analyze motor coordination. The motor coordination variables were two temporal variables (grip to load force onset latency and time to peak grip force) and two force variables (grip force at onset of load force and peak grip force). Functional motor skills were assessed using the Fine Motor Age Equivalents of the Vineland Adaptive Behavior Scale and the Mullen Scales of Early Learning. Mixed regression models were used for all analyses. Children with ASD presented with significant motor coordination deficits only on the two temporal variables, and these variables differentiated children with ASD from the children with TD, but not from children with DD. Fine motor functional skills had no statistically significant associations with any of the motor coordination variables. These findings suggest that subtle problems in the timing of motor actions, possibly related to maturational delays in anticipatory feed-forward mechanisms, may underlie some motor deficits reported in children with ASD, but that these issues are not unique to this population. Further research is needed to investigate how children with ASD or DD compensate for motor control deficits to establish functional skills.

## Introduction

Autism Spectrum Disorders (ASD) are a group of developmental disorders that can cause significant social, communication, and behavioral delays. The development of motor function in persons with ASD is not well understood. Although not usually considered core symptoms of ASD, a variety of unusual motor features are prevalent in this population and are thought to interfere with adaptive behavior (Leary and Hill, 1996; Filipek et al., 1999; Baranek et al., 2005; Mostofsky et al., 2006; Fournier et al., 2010). Estimates of prevalence of motor abnormalities in persons with ASD are upwards of 85% in some studies (Wing, 1981; Miyahara et al., 1997; Provost et al., 2007; Green et al., 2009). Berkeley et al. (2001) found that 50–73% of children with ASD had significant motor delays compared to normative data. Fournier and colleagues presented a meta-analysis of 41 motor coordination studies conducted with children with ASD from 1980 to 2009 and found motor coordination deficits to be a cardinal feature of ASD (Fournier et al., 2010). Some theories on the neurological basis of ASD propose cerebellar abnormalities (Courchesne et al., 1994; Hardan et al., 2001) and establish an association between cerebellar abnormalities and motor abnormalities such as dyscoordination (Muller and Dichgans, 1994; Serrien and Wiesendanger, 1999a,b; Fellows et al., 2001).

Grasping is a fundamental motor activity and is used as a vital mode of exploration for children as they learn about the physical world. Typically, grasping becomes volitional by 4 months of age. Disturbances in grasping patterns may impact how children play, explore, use tools, and engage socially. Provost et al. (2007) noted that motor play activities provide the backdrop for young children to practice social skills and interactions. Leary and Hill (1996) suggested that movement disturbances may impact core ASD symptomology, including social interaction patterns, and communication stating that “the socially referenced core characteristics of autism (e.g., DSM-IV) may be based in part on the presence of neurological symptoms affecting movement” (Leary and Hill, p. 45). Donnellan et al. (2010) distinguished volitional movement deficits as a subset of movement symptoms that particularly affect motivation to move and interest in movement-based environmental exploration.

Research indicates that children with ASD exhibit motor difficulties for simple volitional reach-to-grasp sequences (Hughes, 1996; Mari et al., 2003). Mari et al. (2003) described the importance of reach-to-grasp movements as indicators of neural development. They also suggested that the vast amount of cortical resources devoted to hand coordination functions attests to the functional importance of volitional hand actions. Moreover, in their study of 7–12 years-old children with ASD, Mari et al. (2003) noted variation in the reach-to-grasp performance between high and low intellectual ability (IQ) groupings, suggesting that cognitive maturation may be an important factor in skilled movement and that more research was needed to determine the extent to which cognitive deficits impact movement patterning. Fabbri-Destro et al. (2009) also noted parallels between cognitive deficits and motor deficits in children with ASD during a reach-to-grasp task. Participants were required to reach and place an object in variously sized containers that challenged accuracy requirements. When accuracy demands increased, the children with typical development (TD) presented with reduced reaching and placing speeds, whereas the children with ASD showed reduced placing speeds with no change in reaching speeds. Fabbri-Destro and colleagues concluded that children with ASD tended to program discrete motor acts independently rather than together in a global fashion. They concluded that this could indicate cognitive deficits related to global planning of motor actions.

Although motor disturbances associated with ASD are widely noted, additional investigation of the motor planning and coordination abilities of children with ASD is warranted, particularly with studies containing comparison groups of children with other developmental disabilities (DD). Only one study to date, [i.e., Provost et al. (2007)] has used a group of children with DD as controls. They found that ASD and DD groups do not differ significantly with respect to motor delays on standardized developmental tests. However, they did not investigate grasping specifically nor did they conduct experimental motor control tasks to objectively quantify motor function; thus, more experimental research is needed to better delineate the motor profiles of children with ASD.

Precision grip (i.e., index finger opposed to the thumb to lift an object) is fundamental to overall fine motor functioning. It is relatively simple to perform (typically present by 10 months of age), and experimental tasks of precision grip provide objective quantification of fine motor coordination. Since the cognitive demands of a precision grip task are minimal, very young children or children with lower cognitive or receptive language abilities can be successfully taught to perform a precision grip. Such a task involves first gripping the object (such as a block) using thumb to index finger opposition, and then lifting it off the supporting surface, usually for the purpose of a further volitional action (e.g., in-hand manipulation, placement of object, etc.), (Forssberg et al., 1991). In a precision grip task, there are two forces of interest for coordination—grip force and load force. The gripping force acts perpendicular to the contact surface, while the loading force acts parallel to the contact surface. The latency between the onset of the grip and load force is a measure of coordination (Forssberg et al., 1991). In a well-coordinated execution of the precision grip task, the latency between the onset of grip and load forces are reduced and grip and load forces are programmed in a parallel fashion. In addition, when the precision grip is executed efficiently, the grip force at load force onset is just sufficient to initiate object lift-off and the peak grip forces are scaled such that they are adequate to prevent slippage of the object (Forssberg et al., 1992). Also, the time to achieve peak grip force is indicative of anticipatory feed-forward control. When anticipation of the load and frictional properties of the object are accurate, the time to peak grip force is shortened and the grip force rate is increased when compared to inaccurate anticipation (Forssberg et al., 1992).

Previously, David and colleagues (2009) showed that during a precision grip task, the latency between gripping (grip force) and lifting (load force) an object, and the grip force at onset of load force were significantly increased in children and adolescents with ASD compared to age and sex matched peers with TD. Given the older age of this sample, it is unclear if these motor deficits were the result of aberrant developmental mechanisms and/or the progressive lack of experience with functional motor skills. There is a dearth of literature utilizing controls with DD to enable identification of motor characteristics unique to ASD, particularly very early in development. Thus, more studies using both TD and DD comparison groups, across wider age ranges and cognitive levels are needed to determine the pathogenesis of motor deficits in ASD, as well as to potentially facilitate differential diagnosis and intervention planning.

The current study employed the precision grip task used by David et al. (2009) and addressed limitations in the literature by (1) including a comparison group of children with DD matched on chronological age (CA) and mental age (MA) in order to isolate findings that might be unique to ASD, and separate from intellectual disability, (2) analyzing the development of grasp using a cross-sectional methodology with a younger sample (i.e., children ages 2–6 years) than previously conducted, and (3) investigating the potential associations of experimental measures with standardized assessments of motor development.

Specifically, this cross-sectional quasi-experimental study aimed to:

1. Describe developmental correlates of motor coordination during a grasping task in children with ASD (2–6 years). Given that motor functioning deficits in older children with ASD are associated with cognitive deficits (Mari et al., 2003), we hypothesized that MA would be a stronger correlate than CA for the children with ASD, as well as for children with DD.
2. Identify the extent to which motor coordination deficits are unique to ASD. Given that David et al. (2009) reported coordination deficits (i.e., increased latency between grip and load force; increased grip force at load force onset) in older children with ASD, we hypothesized that that children with ASD would have significantly less motor coordination than the other two groups during the precision grasping task for both force and temporal variables.
3. Determine the association between the experimental motor coordination variables and functional fine motor skills. Because precision grip is integral to so many fine motor functional skills, we hypothesized that deficits on the precision grip task would predict greater impairments in functional motor skills on standardized developmental measures.

## Materials and methods

### Participants

Participants were recruited through a collaborating NIH grant-funded project, an autism research registry, and various community agencies. We attempted to recruit age ranges equally represented across the three groups. Each group was stratified into ages: 2–3 years, 3–4 years, and 4–6 years. Based on our previous findings, a power analysis estimated the power to range between 0.95 and 0.99 for a sample size of 21 per diagnostic group for grip to load force onset latency and grip force at onset of load force.

Inclusion criteria for children with ASD included (1) a diagnosis of Autistic Disorder (American Psychiatric Association, 1994) from a licensed professional (psychologist or physician), confirmed by results of the Autism Diagnostic Interview-Revised (ADI-R; Lord et al., 1994) and the Autism Diagnostic Observation Schedule (ADOS; Lord et al., 1999), (2) no known genetic/medical conditions strongly associated with ASD (e.g., fragile × syndrome; tuberous sclerosis) as confirmed by medical records/examinations, (3) normal or corrected hearing and vision, (4) no musculoskeletal defects that may prevent completion of the grasping task, and (5) no psychoactive medications that might produce motor side effects (Advokat et al., 2000).

Inclusion criteria for children with DD included (1) confirmed DD associated with intellectual delay and those with non-specific developmental delays that demonstrated delays of at least −1.5 standard deviations in at least two areas of development (i.e., Expressive Language, Receptive Language, Cognitive/Visual Reception, Fine or Gross Motor, and/or Adaptive behavior) confirmed by developmental testing (Leiter International Performance Scale-Revised—LIPS-R; Roid and Miller, 1997; or Mullen Scales of Early Learning—MSEL; Mullen, 1995; and Vineland Adaptive Behavior Scale—VABS; Sparrow et al., 1984), (2) autism status ruled out by ADOS, (3) no genetic or medical conditions with well-documented increased co-morbidity with ASD, (4) normal or corrected hearing and vision, (5) no musculoskeletal defects that may prevent completion of the grasping task, and (6) no psychoactive medications.

Inclusion criteria for children with TD included those with (1) scores within the average or above range on the Leiter-R or Mullen Scales, and VABS, (2) normal or corrected hearing and vision, and (3) no musculoskeletal defects that may prevent completion of the grasping task. Excluded from the TD group were any children (1) whose parents expressed significant concerns about their development, (2) with a history of developmental problems, and (3) who received special education or related therapeutic services (e.g., speech-language therapy). In addition, each child in the group with TD was screened for autistic symptoms with the Childhood Autism Rating Scale (CARS; Schopler et al., 1986) and excluded if symptoms of autism were noted using a conservative cut point (i.e., score >25).

### Materials

**Figure 1:**
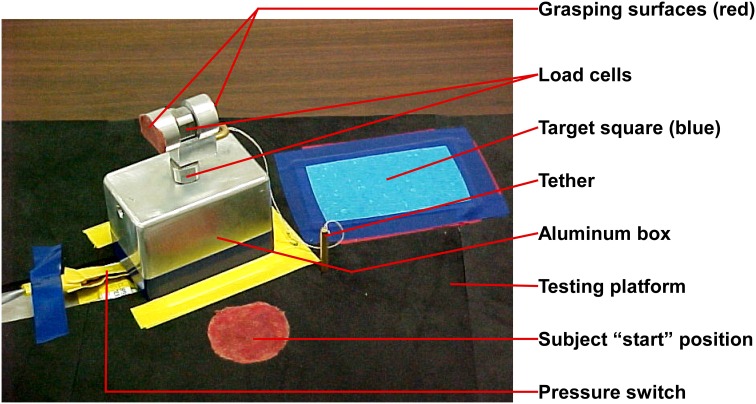
**Grasping apparatus**.

The experimental apparatus (Figure 1) was similar to that described by David et al. (2009). It had two orthogonally placed load cells; one measured grip force, while the other measured load force. The loads were suspended within an aluminum box, and size cues were invariant between loads. For this study with younger children, the design of the experimental grasping apparatus was modified to be more child-friendly in appearance and lighter in total weight (weight not totaling more than 16 oz., which included the added Newton weights) in order to facilitate lift. We used individualized age-appropriate visual stimuli (e.g., stickers), that were stuck on to the apparatus and on to the target square, to optimize motivation. The equipment was portable and the majority of the data were collected at the university research facilities with a few testing sessions occurring in participant's homes.

### Procedures

This study was approved by the institutional review board. A letter describing the study was mailed to parents with study team contact information. Interested parents contacted the study coordinator, oral consent was obtained, a preliminary eligibility form was completed via phone, and an appointment for experimental testing in the laboratory was scheduled. Written consent was obtained from all parents of all children who participated in the study. Parents were paid $12.50 per hour up to $50 for their child's participation in the assessments and grasp testing over a 2-day testing period.

During experimental testing parents and children completed several assessments. All of the assessments were valid for children in the chronological and developmental age range of interest, and demonstrated good psychometric properties. The following three assessments were used to confirm the diagnosis of ASD. (1) The ADI-R (Lord et al., 1994), a semi-structured parent interview that is the gold-standard diagnostic measure based on the diagnostic criteria for autism in the Diagnostic and Statistical Manual of Mental Disorders (American Psychiatric Association, 1994). (2) The ADOS (Lord et al., 1999), an observational assessment designed to assess the presence and severity of symptoms. (3) The CARS (Schopler et al., 1986), a 15-item behavioral rating scale that was used to screen for the presence of autistic symptoms. The ADI-R and ADOS were administered only to the children with ASD and DD. The CARS was administered to all children during the clinical assessments.

The following two scales were used to rate cognitive ability. (1) The LIPS-R (Roid and Miller, 1997) is a non-verbal measure of intelligence, well-suited for children impaired in their ability to respond on verbal tests and was used for children with MAs below 2 years. We used the “Brief IQ,” a valid measure of cognitive abilities, which is based on four subtests of the Visualization and Reasoning Battery (Repeated Patterns, Sequential Order, Figure-Ground, and Form Completion). MA was generated by the software program using the raw scores, IQ, and age, and these were used as the developmental variable in the analyses. (2) The MSEL (Mullen, 1995) is a comprehensive measure of development for infants and preschool children from birth to 68 months and contains four subscales that were administered (Visual Reception, Expressive Language, Receptive Language, and Fine Motor). The Visual Reception scale is a valid measure of cognitive abilities that is not confounded by verbal or motoric demands. The MSEL Visual Reception scores or the LIPS-R MA equivalents were used for purposes of matching between the groups with ASD and DD, and as a measure of MA in analyses. The MSEL Fine Motor scale was used as a measure of functional fine motor abilities.

Adaptive behavior was rated using the VABS (Sparrow et al., 1984), a well-standardized and norm-referenced structured parent interview designed to evaluate children's (0–18 years) adaptive behavior in four areas (communication, daily living skills, socialization, and motor skills). This instrument was completed for all children. Fine motor age equivalent scores were calculated and used in the analyses as a measure of functional motor skills.

Handedness was rated using the Edinburgh Handedness Inventory (Oldfield, 1971), a parent questionnaire. Only those items suitable for the developmental age range in the study were used. If the results of the inventory showed mixed dominance, then the hand used for self-feeding with a spoon was used as the dominant hand.

All parents completed a demographics questionnaire, the Edinburgh Handedness Inventory (Oldfield, 1971), and the VABS (Sparrow et al., 1984). Parents of children with ASD were also administered the ADI-R (Lord et al., 1994). All children were rated on the LIPS-R (Roid and Miller, 1997), the MSEL (Mullen, 1995), and the CARS (Schopler et al., 1986). Children with ASD and DD were also rated on the ADOS (Lord et al., 1999). All observational assessments were administered in a child friendly laboratory and children were given breaks and self-selected reinforcers as needed.

The children in the groups with ASD and DD were matched on gender, CA and MA. The group with TD was matched on CA and gender to the group with ASD. MA was not used as a matching criterion because, given that the task was a motor task, and given that coordinations of grip and load begins to emerge only by the age of two (Eliasson et al., 1995), matching the TD group with the ASD group on MA would have created a very young group with TD and would have resulted in a developmental disadvantage for the group with TD.

**Table:** **Panel: Abbreviations**.

**DIAGNOSTIC GROUPS**			
Abbreviation	Full Name		
ASD	Autism Spectrum Disorders		
DD	Developmental Delay		
TD	Typical Development		
**ASSESSMENT MEASURES**			
Abbreviation	Full Name	Authors	Used for
ADI-R	Autism Diagnostic Interview-Revised	Lord et al., 1994	confirmation of ASD diagnosis
ADOS	Autism Diagnostic Observation Schedule	Lord et al., 1999	confirmation of ASD diagnosis
CARS	Childhood Autism Rating Scale	Schopler et al., 1986	screening for autistic symptoms
LIPS-R	Leiter International Performance Scale-Revised	Roid and Miller, 1997	Brief IQ scores used as measure of cognitive ability to generate mental age
MSEL	Mullen Scales of Early Learning	Mullen, 1995	Visual Reception scores used as measure of cognitive ability to generate mental age
VABS	Vineland Adaptive Behavior Scale	Sparrow et al., 1984	assessment of functional fine motor skills
	Edinburgh Handedness Inventory	Oldfield, 1971	identification of hand dominance
**MOTOR COORDINATION VARIABLES**			
Abbreviation	Full Name	Unit of measurement	Description
GLOT	grip to load force onset laterncy	miliseconds (ms)	temporal variable: time between beginning to grip and beginning to lift object
tPGE	time to peak grip force	miliseconds (ms)	temporal variable: time between beginning to grip an object and the point of maximal (tightest) grip
GFATLF	grip force at onset of load force	Newtons (N)	force variable: tightness of grip when starting to lift object
PGF	peak grip force	Newtons (N)	force variable: maximal tightness of grip during the task

Children were seated comfortably at a testing table with back and feet supported in a height adjustable chair. The chair was adjusted so that the height of the table was 3–5′ above the elbow (Bendix, 1987). An alternative position for younger children was to be seated on their caregiver's lap at a table. The experimental apparatus was placed on the testing table in front of the child at her/his midline at a distance equivalent to 60% of the child's arm length (Kuhtz-Buschbeck et al., 1998). Arm length was defined as the distance between the acromion and the radial styloid of the dominant arm (Kuhtz-Buschbeck et al., 1998). The child placed her/his dominant hand on a “start” position which was marked using red adhesive tape to ensure procedural fidelity and located 5 cm posterior to the experimental apparatus. The non-dominant hand was placed below the table on the child's ipsilateral thigh. Speed of the movement was self-selected. The children were instructed orally and using investigator demonstration to lift the apparatus using a precision grasp. A precision grasp was operationally defined as a grasp that involves using the thumb and two or more of the remaining fingers without the object contacting the volar/palmar surface of the hand.

Simple directions were given—“Pick up ‘name of object’ (e.g., car, puppy, and Sponge Bob sticker that was stuck on to the experimental apparatus, etc.) and put on the picture of ‘name of same object’ that you see on the table.” Upon hearing the verbal cue, “Go” the child grasped the apparatus, lifted it off the testing platform, and placed it on the target area (brightly colored square with picture of same object). The instructions were modified, and demonstration and physical cues were provided as needed. Data collection encompassed the duration from the child's initial contact with the apparatus to the apparatus lift-off from the supporting surface.

We recorded grip and load force of each participant as they grasped and lifted the experimental apparatus. Two circular load cells, (Kistler Instrumentation Corporation) placed orthogonal to each other on the apparatus, simultaneously recorded grip and load forces. The weight of the experimental apparatus was 3.6N. The children were blinded to the insertion of pre-calibrated Newton weights (0.5N, 2N, and 1N) into the experimental apparatus. Therefore, for Load 1 the total weight was 4.1N, Load 2 total weight was 5.6N, and Load 3 total weight was 4.6N. Each participant performed a total of two practice trials for each load category. This was followed by test trials, which were three blocks of five trials, one block for each load category. The order of presentation of added weight to the apparatus was 0.5N-2N-1N (light-heavy-light). The standardized order of presentation was designed to discern the effect of anticipation with alternating loads (light-heavy-light) to be used in future analyses. Data were recorded on a laptop computer. During the trials, if the research assistant observed that the participant (1) failed to use a precision grip, or (2) failed to grasp the apparatus on the grasping surfaces a “mistrial” was designated and the participant repeated the trial. The experimental task took at average of 40 min to complete.

### Data reduction

Analog data were sampled at 125 Hz. The duration of each trial ranged between 0.5 and 3 s. The analog data were amplified using a charge amplifier (0.1 volt represented 1N), converted from analog to digital using an analog-to-digital converter, and digitally smoothed using a 10 Hz Butterworth low-pass filter. The force signals were processed using a custom written program in Matlab 7(R14) (The Mathworks Inc, 2004). Motor coordination was measured using two temporal variables, i.e., grip to load force onset latency and time to peak grip force, and two force variables, i.e., grip force at onset of load force and peak grip force.

Grip to load force onset latency was defined as the duration between onset of grip force and onset of a load force. Time to peak grip force was defined as the duration between the onset of grip force and maximum amplitude of grip force. Grip force at onset of load force was defined as the amplitude of grip force at onset of load force. Peak grip force was defined as the maximum amplitude of the grip force profile.

### Statistical analysis

#### Aim 1 and 2

Mixed regression modeling (SAS 9.1) was used to address the cross sectional effect of age on the motor coordination variables (i.e., grip to load force onset latency, time to peak grip force, grip force at onset of load force, and peak grip force) and to identify trends that were unique to the group with ASD relative to the group with DD and TD. CA and MA were analyzed in separate models. Thus, two mixed regression models were used for each motor coordination variable. Neither model used load as a predictor because preliminary analyses revealed no effect of load. The first model examined the effect of group, CA, and group by CA interactions. The second model examined the effect of group, MA, and group by MA interactions. The “general” full model is given below

$${\text{Y}}_{ij}=\beta_{0}+\beta_{1}{\text{Group}}_{i}+\beta_{2}{\text{Age}}_{i}+\beta_{3}{\text{Group}}_{i}\times {\text{Age}}_{i}+\nu_{0i}+\varepsilon_{ij}$$

where, Y is the motor coordination variable (grip to load force onset latency or grip force at onset of load force or peak grip force or time to peak grip force) for the *i*^th^ individual for the *j*^th^ load

β_0_ is the intercept
β_1_ is the effect of group
β_2_ is the effect of Age (CA or MA)
β_3_ is the interaction between group and age (CA or MA)
ν_0_ is the individual's influence on repeated observation for the different load categories
ε is the error term.

#### Aim 3

Mixed regression modeling (SAS 9.1) was used to analyze the relationship between fine motor functional skills and motor coordination variables. Fine motor functional skills were quantified using the VABS (a parent report) and MSEL (rated by a trained observer) fine motor age equivalents. Thus the full model included group, VABS and MSEL fine motor age equivalents, and group by fine motor function interaction terms. The “general” full model is given below:

$${\text{Y}}_{ij}=\beta_{0}+\beta_{1}{\text{Group}}_{i}+\beta_{2}{\text{VABS}}_{i}+\beta_{3}{\text{MSEL}}_{i}+\beta_{4}{\text{Group}}_{i}\times {\text{VABS}}_{i}+\beta_{5}{\text{Group}}_{i}\times {\text{MSEL}}_{i}+\beta_{6}{\text{Group}}_{i}\times {\text{VABS}}_{i}\times {\text{MSEL}}_{i}+\nu_{0i}+\varepsilon_{ij}$$

where, Y is the motor coordination variable (grip to load force onset latency or grip force at onset of load force or peak grip force or time to peak grip force) for the *i*^th^ individual for the *j*^th^ load

β_0_ is the intercept
β_1_ is the effect of group
β_2_ is the effect of the VABS fine motor age equivalent
β_3_ is the effect of the MSEL fine motor age equivalent
β_4_ is the group by VABS fine motor age equivalent interaction
β_5_ is the group by MSEL fine motor age equivalent interaction
β_6_ is the group by VABS by MSEL fine motor age equivalent score interaction
ν_0_ is the individual's influence on repeated observation for the different load categories
ε is the error term.

## Results

Of the 83 children recruited and tested, only 65 had valid data on the motor coordination variables. Only the data from these 65 children are included in this paper. Demographic and clinical details are reported in Table 1. The group with ASD had a mean age of 54 months (*SD* = 13; min–max = 31–76), the group with DD had a mean age of 54.5 months (*SD* = 15.6; min–max = 25–77), and the group with TD had a mean age of 47.3 months (*SD* = 18.8; min–max = 20–77). The composition of the three groups varied across several variables. All groups had higher percentages of male participants, although the group with TD had a relatively greater proportion of female to male participants compared to the other two groups. Although there are no studies comparing fine motor coordination between boys and girls with ASD, there are documented sex differences in maximal grip strength; however, maximal grip strength is unlikely to be a factor that affected our results because the force required to lift the object was well within the maximal grip strength of the participants.

**Table 1:** **Participant demographics**.

	**ASD (*n* = 24)**	**DD (*n* = 11)**	**TD (*n* = 30)**
**DEMOGRAPHIC CHARACTERISTICS**			
**Sex – n (%)**			
Female	3 (12.5)	3 (27.3)	13 (43.4)
Male	21 (87.5)	8 (72.7)	17 (56.7)
**Age – mean (SD)**			
CA in months	54.0 (13.0)	54.5 (15.6)	47.3 (18.8)
CA min–max	31.0–76.0	25.0–77.0	20.0–77.0
**Ethnic category – n (%)**			
Hispanic or latino	1 (4.5)	0 (0)	10 (30)
Not hispanic or latino	21 (94.5)	11 (100)	20 (70)
Missing	2 (8.3)	0 (0)	0 (0)
**Race – n (%)**			
Asian or pacific Islander	0	2 (18.2)	3 (10)
Black or African American	3 (12.5)	1 (9.1)	1 (3.3)
White	19 (79.2)	8 (72.7)	23 (76.7)
Other	1 (4.1)	0 (0)	0 (0)
Unknown	1 (4.1)	0 (0)	0 (0)
**Mother's education – n (%)**			
High school diploma or less	4 (16.7)	2 (18.2)	1 (3.3)
Some college or AA	7 (29.2)	1 (9.1)	1 (3.3)
BA/BS	8 (33.3)	3 (27.3)	14 (46.7)
MA/MS+	5 (20.8)	5 (45.5)	13 (43.3)
Missing	0 (0)	0 (0)	1 (3.3)
**CLINICAL CHARACTERISTICS**			
**Mental age – mean (SD)**^†^			
MA in months	31.8 (14.1)	44.3 (18.1)	48.6 (16.1)
MA min–max	9.0–69.0	17.0–69.0	23.0–69.0
**VABS – mean (SD)**			
Adaptive behavior composite- age equivalents in months	26.4 (12.4)	33.4 (11.0)	48.8 (18.5)
Fine motor – age equivalents in months	32.7 (13.4)	39 (18.7)	41.1 (17.2)
**MSEL – mean (SD)**			
Fine motor – age equivalents in months	32.2 (14.2)	36.6 (11.2)	46.1 (16.9)
CARS – mean (SD)	34.7 (7.8)	20.6 (4.0)	15.5 (0.6)

ASD, Autism Spectrum Disorders; DD, Developmental Delay; TD, Typical Development; SD, Standard Deviation; CA, Chronological Age; MA, Mental Age; LIPS, Leiter International Performance Scale; MSEL, Mullen Scales of Early Learning; VABS, Vineland Adaptive Behavior Scale; CARS, Childhood Autism Rating Scale.

† MSEL, visual reception subscale was used for children ≤ 68 months. LIPS was used for children > 68 months.

**Figure 2:**
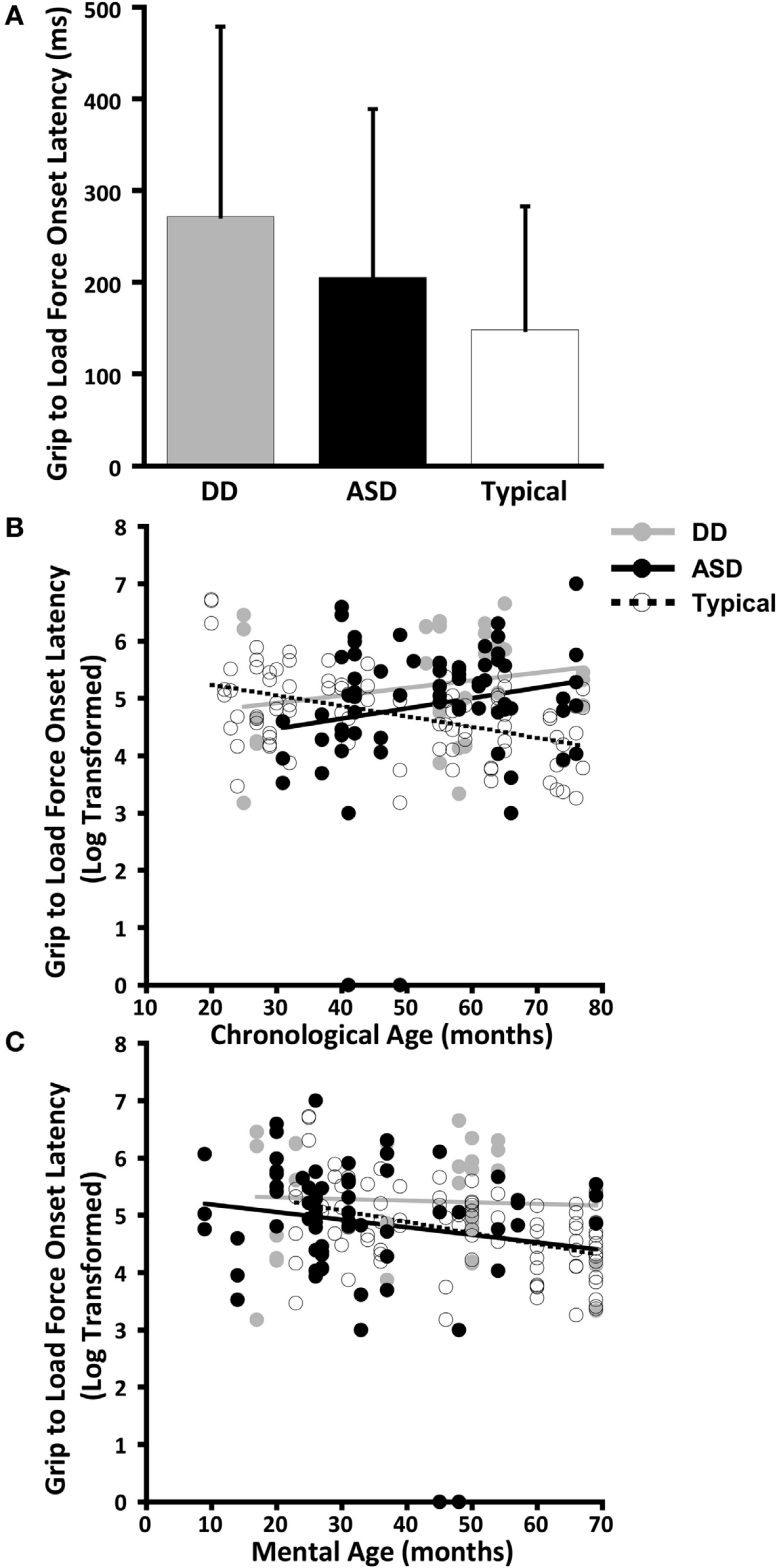
**Grip to load force onset latency. (A)** Means and standard deviations. **(B)** The effect of chronological age. **(C)** The effect of mental age.

**Figure 3:**
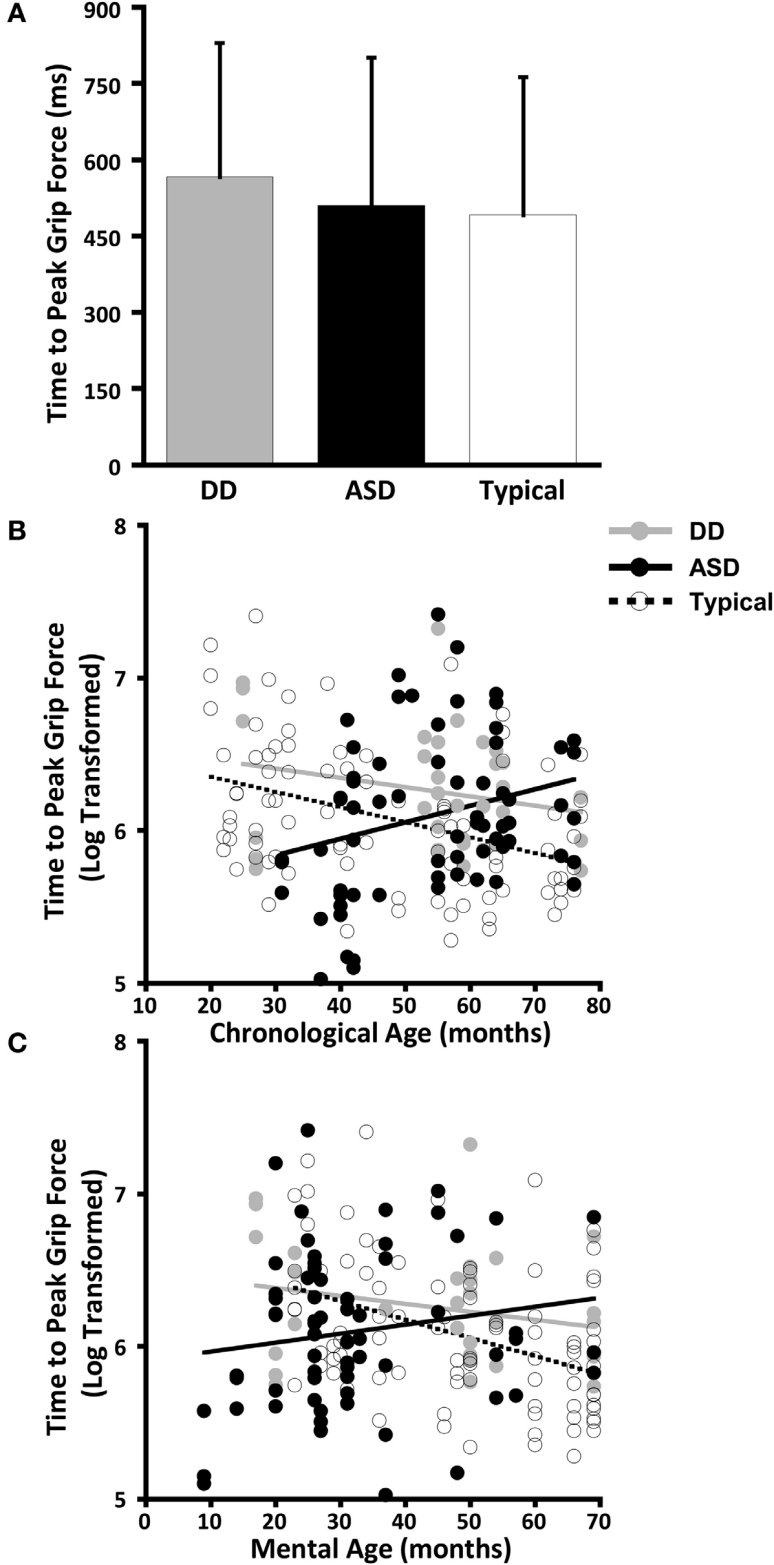
**Time to peak grip force. (A)** Means and standard deviations. **(B)** The effect of chronological age. **(C)** The effect of mental age.

**Figure 4:**
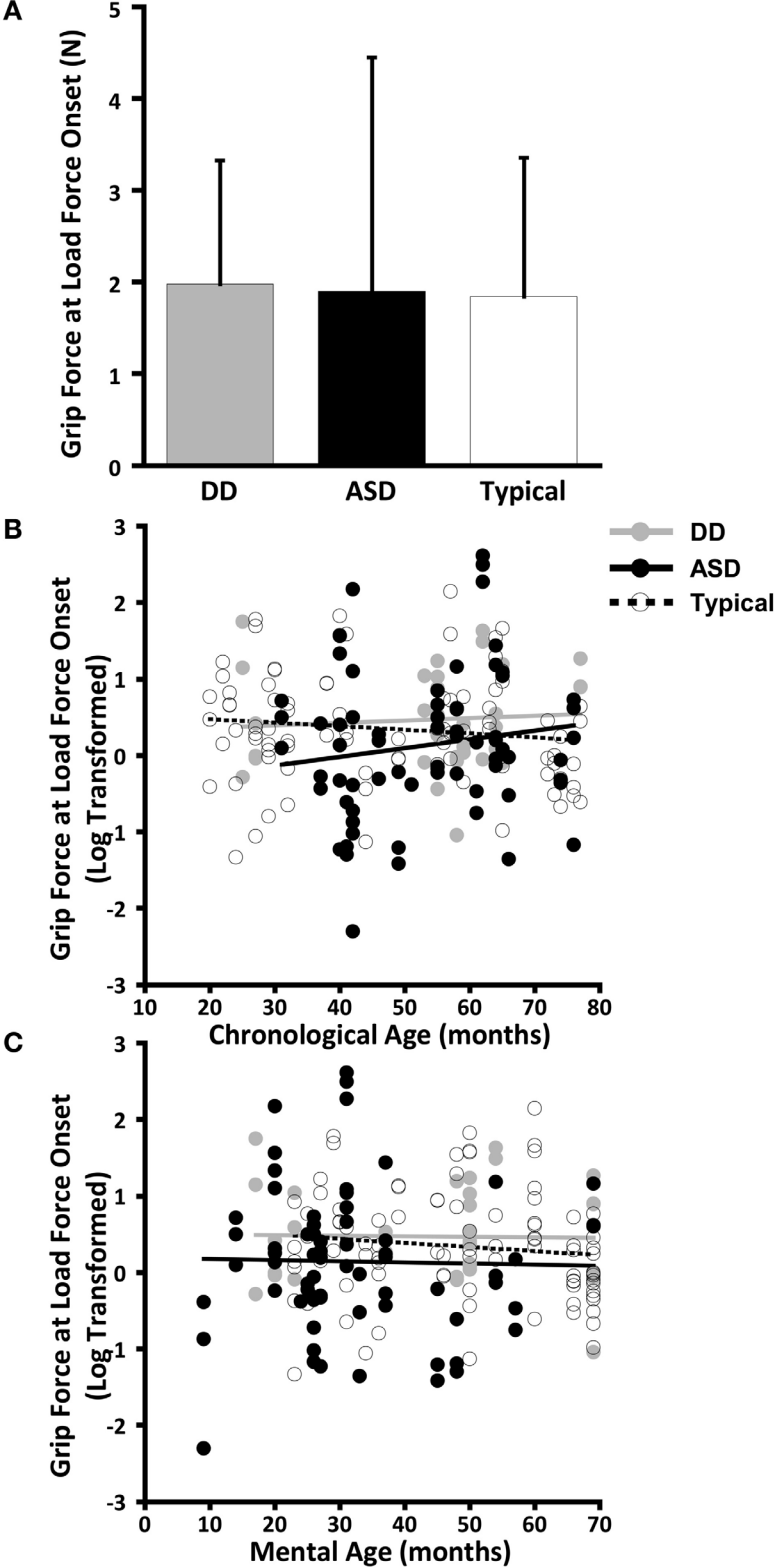
**Grip force at onset of load force. (A)** Means and standard deviations. **(B)** The effect of chronological age. **(C)** The effect of mental age.

**Figure 5:**
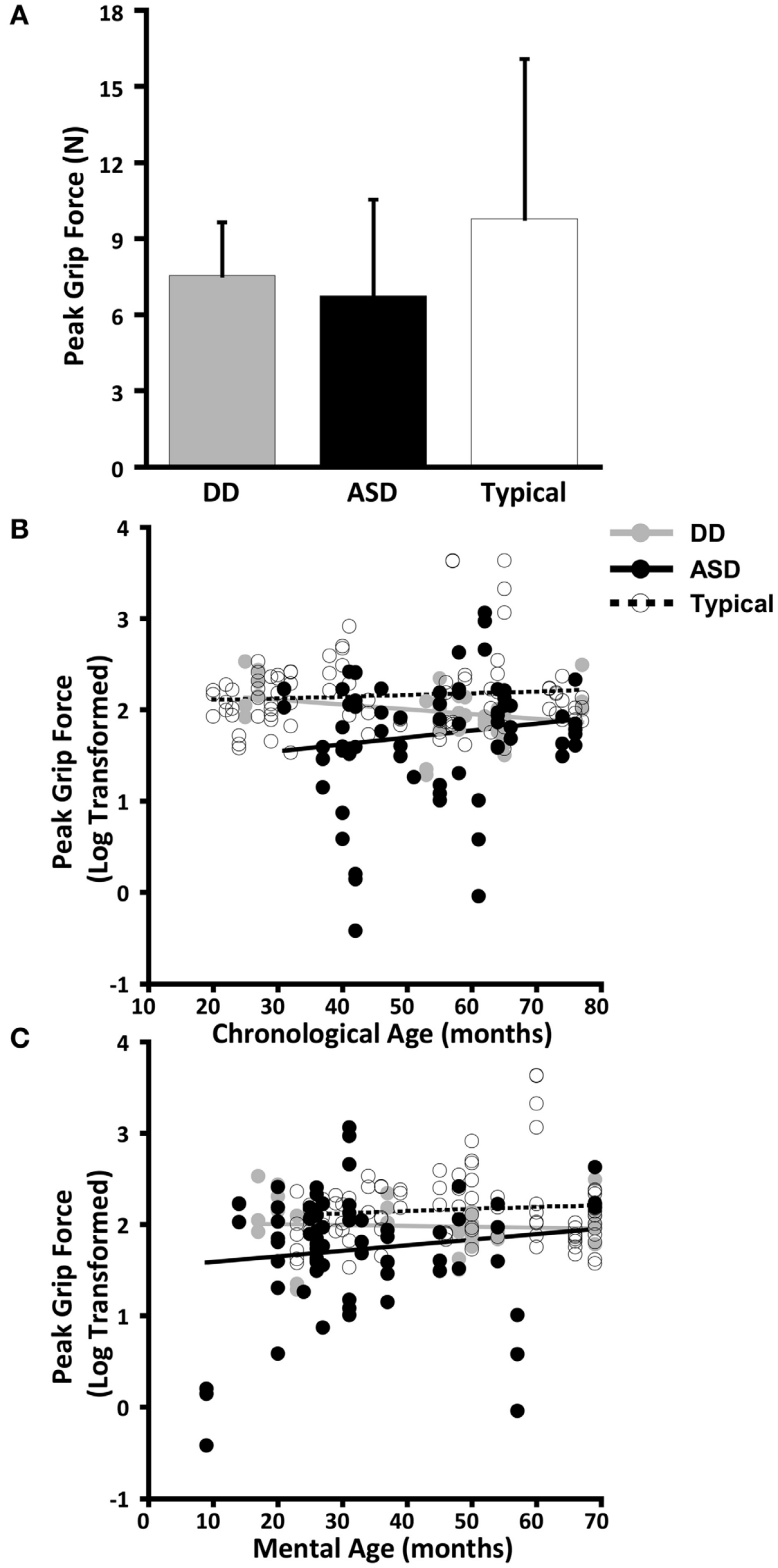
**Peak grip force. (A)** Means and standard deviations. **(B)** The effect of chronological age. **(C)** The effect of mental age.

The dependent variables did not meet the distributional assumptions required for the mixed model regression analysis and were log transformed. Table 2 and Figures 2A, 3A, 4A, and 5A lists the means and standard deviations for each motor coordination variable (i.e., grip to load force onset latency, grip force at onset of load force, peak grip force, and time to peak grip force) by load across each diagnostic group for the untransformed data.

**Table 2:** **Motor coordination variables across participants**.

**Group**	**Load (*N*)**	**GLOT (ms)**		**tPGF (ms)**		**GFATLF (N)**		**PGF (N)**	
		**Mean**	**SD**	**Mean**	**SD**	**Mean**	**SD**	**Mean**	**SD**
ASD (*n* = 24)	0.5	209.51	159.87	528.90	301.92	1.77	2.00	6.65	3.17
	1	215.82	231.13	496.35	355.19	1.75	2.47	6.21	3.74
	2	190.86	159.56	504.29	207.86	2.17	3.18	7.35	4.55
	Mean	205.52	183.33	510.41	290.82	1.90	2.55	6.73	3.81
DD (*n* = 11)	0.5	264.91	200.99	627.73	357.23	1.81	1.13	7.28	1.39
	1	289.09	196.85	571.82	198.86	1.80	1.10	6.88	1.66
	2	262.00	240.22	499.27	214.01	2.32	1.77	8.50	2.78
	Mean	272.00	207.17	566.27	263.44	1.98	1.35	7.55	2.09
TD (*n* = 30)	0.5	143.17	110.51	469.80	209.82	1.80	1.51	10.01	6.98
	1	148.97	145.56	515.77	295.47	1.80	1.63	9.36	6.24
	2	151.72	150.51	487.00	308.36	1.93	1.47	9.97	5.81
	Mean	147.91	135.01	490.90	271.85	1.84	1.52	9.78	6.30

N, Newtons; GLOT, grip to load force onset latency; ms, milliseconds; tPGF, time to peak grip force; GFATLF, grip force at onset of load force; PGF, peak grip force; SD, standard deviation; ASD, Autism Spectrum Disorders; DD, Developmental Delay; TD, Typical Development.

### Aim 1 and 2: the effect of chronological age and mental age on motor coordination variables and identification of characteristics unique to the group with ASD

As a general rule, in the event of a significant interaction, the main effects are un-interpretable. This is because the main effect of group or differences between groups is a conditional effect, and is only applicable for a specific CA or MA. For instance, for time to peak grip force, significant interactions between MA and group indicate that the differences between the groups vary as a function of MA. To elaborate, when MA = 9 (the minimum MA in our sample), the time to peak grip force of the ASD group was shorter than the TD group (*p* = 0.01) and not different from the DD group (*p* = 0.09). However, when MA = 69 (the maximum MA in our sample), the time to peak grip force of the ASD group was longer than the TD group (*p* = 0.04) and not different from the DD group (*p* = 0.51). Therefore, if a significant interaction is observed, then main effects will not be addressed.

#### Grip to load force onset latency

The effect of CA was significantly different between the group with ASD and the group with TD (*P* = 0.005) but not between the group with ASD and the group with DD (*P* = 0.8) (Table 3, Figure 2B). In our cross-sectional sample, as children with TD got older, their grip to load force onset latency decreased (Figure 2B, dashed black line). However, this was not the case in the groups with ASD or DD, i.e., changes in grip to load force onset latency were not associated with changes in CA (Figure 2B, solid black and grey lines).

**Table 3:** **Grip to load force onset latency and chronological age (CA)**.

**Effect**	**Estimate**	**Standard error**	**DF**	***t*-value**	***p***
**MAIN EFFECTS**					
CA					0.528
Group					0.042^*^
Interaction					0.006^*^
***POST-HOC***					
**Group**					
TD vs. ASD	1.66	0.68	124.00	2.44	0.016^*^
DD vs. ASD	0.63	0.96	124.00	0.66	0.510
DD vs. TD	−1.02	0.83	124.00	−1.24	0.216
**Interaction**					
CA effect: TD vs. ASD	−0.04	0.01	124.00	−2.88	0.005^*^
CA effect: DD vs. ASD	−0.004	0.02	124.00	−0.26	0.8
CA effect: DD vs. TD	0.03	0.01	124.00	2.13	0.035^*^

TD, Typical Development; ASD, Autism Spectrum Disorders; DD, Developmental Delay.

* P-value < *0.05*.

MA was a significant predictor of onset latency (*P* = 0.024). As MA increased the grip to load force onset latency decreased. There was no group effect (*P* = 0.777), nor was there a group by MA interaction (*P* = 0.636) (Table 4, Figure 2C). Thus, the effect of MA was similar between children with ASD, DD, and TD, and averaged across the three groups was a significant predictor of grip to load force onset latency.

**Table 4:** **Grip to load force onset latency and mental age (MA)**.

**Effect**	**Estimate**	**Standard error**	**DF**	***t*-value**	***p***
**MAIN EFFECTS**					
MA					0.024^*^
Group					0.777
Interaction					0.636
***POST-HOC***					
**Group**					
TD vs. ASD	0.363	0.536	124.00	0.68	0.5
DD vs. ASD	0.278	0.630	124.00	0.44	0.660
DD vs. TD	−0.08	0.67	124.00	−0.13	0.90
**Interaction**					
MA effect: TD vs. ASD	−0.006	0.01	124.00	−0.5	0.617
MA effect: DD vs. ASD	−0.007	0.02	124.00	0.43	0.666
MA effect: DD vs. TD	0.01	0.01	124.00	0.94	0.351

TD, Typical Development; ASD, Autism Spectrum Disorders; DD, Developmental Delay.

* P-value < *0.05*.

#### Time to peak grip force

The effect of CA was significantly different between the groups with ASD and TD (*P* = 0.004) but not between the groups with ASD or DD (*P* = 0.086) (Table 5, Figure 3B). In our cross-sectional sample as children with TD got older, their time to peak grip force decreased (Figure 3B, dashed black line). A similar effect of CA was observed in the group with DD; however, this effect only approached significance (*P* = 0.086) relative to the group with ASD (Figure 3B, solid black and grey lines).

**Table 5:** **Time to peak grip force and chronological age (CA)**.

**Effect**	**Estimate**	**Standard error**	**DF**	***t*-value**	***p***
**MAIN EFFECTS**					
CA					0.526
Group					0.024^*^
Interaction					0.015^*^
***POST-HOC***					
**Group**					
TD vs. ASD	0.98	0.37	124.00	2.68	0.008^*^
DD vs. ASD	1.03	0.51	124.00	2.0	0.046^*^
DD vs. TD	0.05	0.45	124.00	0.1	0.917
**Interaction**					
CA effect: TD vs. ASD	−0.02	0.01	124.00	−2.94	0.004^*^
CA effect: DD vs. ASD	−0.02	0.01	124.00	−1.73	0.086
CA effect: DD vs. TD	0.004	0.01	124	0.48	0.633

TD, Typical Development; ASD, Autism Spectrum Disorders; DD, Developmental Delay.

* P-value < *0.05*.

The effect of MA was significantly different between the groups with ASD and TD (*P* = 0.013) but not between the groups with ASD and DD (*P* = 0.175) (Table 6, Figure 3C). In our cross-sectional sample as the MA of children with TD increased, their time to peak grip force decreased (Figure 3C, dashed black line). A similar effect of MA was observed in the group with DD; however, this effect was not significantly different from the group with ASD (Figure 3C, solid black and grey lines).

**Table 6:** **Time to peak grip force and mental age (MA)**.

**Effect**	**Estimate**	**Standard error**	**DF**	***t*-value**	***p***
**MAIN EFFECTS**					
MA					0.188
Group					0.028^*^
Interaction					0.044^*^
***POST-HOC***					
**Group**					
TD vs. ASD	0.723	0.284	124.00	2.55	0.012^*^
DD vs. ASD	0.569	0.324	124.00	1.76	0.082
DD vs. TD	−0.15	0.35	124.00	−0.44	0.658
**Interaction**					
MA effect: TD vs. ASD	−0.017	0.01	124.00	−2.53	0.013^*^
MA effect: DD vs. ASD	−0.011	0.01	124.00	−1.36	0.175
MA effect: DD vs. TD	0.006	0.01	124.00	0.88	0.382

TD, Typical Development; ASD, Autism Spectrum Disorders; DD, Developmental Delay.

* P-value < *0.05*.

#### Grip force at load force onset

CA was not a significant predictor of grip force at onset of load force (*P* = 0.642), nor was there an effect of group (*P* = 0.373), nor was there a CA by group interaction (*P* = 0.501). (Table 7, Figure 4B).

**Table 7:** **Grip force at onset of load force and chronological age (CA)**.

**Effect**	**Estimate**	**Standard error**	**DF**	***t*-value**	***p***
**MAIN EFFECTS**					
CA					0.642
Group					0.373
Interaction					0.501
***POST-HOC***					
**Group**					
TD vs. ASD	0.98	0.70	124.00	1.41	0.161
DD vs. ASD	0.72	0.98	124.00	0.74	0.462
DD vs. TD	−0.26	0.85	124.00	−0.31	0.759
**Interaction**					
CA effect: TD vs. ASD	−0.01	0.01	124.00	−1.16	0.25
CA effect: DD vs. ASD	−0.01	0.02	124.00	−0.41	0.68
CA effect: DD vs. TD	0.008	0.02	124.00	0.49	0.623

TD, Typical Development; ASD, Autism Spectrum Disorders; DD, Developmental Delay.

MA results were similar to CA results. MA was not a significant predictor of grip force at onset of load force (*P* = 0.642), nor was there an effect of group (*P* = 0.373), nor was there a MA by group interaction (*P* = 0.501). (Table 8, Figure 4C).

**Table 8:** **Grip force at onset of load force and mental age (MA)**.

**Effect**	**Estimate**	**Standard error**	**DF**	***t*-value**	***p***
**MAIN EFFECTS**					
MA					0.621
Group					0.728
Interaction					0.961
***POST-HOC***					
**Group**					
TD vs. ASD	0.384	0.540	124.00	0.71	0.478
DD vs. ASD	0.344	0.586	124.00	0.59	0.558
DD vs. TD	−0.04	0.643	124.00	−0.06	0.951
**Interaction**					
MA effect: TD vs. ASD	−0.002	0.01	124.00	−0.23	0.822
MA effect: DD vs. ASD	<0.001	0.01	124.00	0.02	0.982
MA effect: DD vs. TD	0.003	0.01	124.00	0.24	0.811

TD, Typical Development; ASD, Autism Spectrum Disorders; DD, Developmental Delay.

#### Peak grip force

CA was not a significant predictor of peak grip force (*P* = 0.852), nor was there an effect of group (*P* = 0.502), nor was there a CA by group interaction (*P* = 0.759). (Table 9, Figure 5B).

**Table 9:** **Peak grip force and chronological age (CA)**.

**Effect**	**Estimate**	**Standard error**	**DF**	***t*-value**	***p***
**MAIN EFFECTS**					
CA					0.852
Group					0.502
Interaction					0.759
***POST-HOC***					
**Group**					
TD vs. ASD	0.55	0.479	124.00	1.14	0.255
DD vs. ASD	0.55	0.667	124.00	0.83	0.411
DD vs. TD	0.003	0.59	124.00	0.01	0.996
**Interaction**					
CA effect: TD vs. ASD	−0.002	0.01	124.00	−0.27	0.788
CA effect: DD vs. ASD	−0.01	0.01	124.00	−0.73	0.466
CA effect: DD vs. TD	−0.007	0.02	124.00	−0.61	0.545

TD, Typical Development; ASD, Autism Spectrum Disorders; DD, Developmental Delay.

MA results were similar to CA results. MA was not a significant predictor of grip force at onset of load force (*P* = 0.218), nor was there an effect of group (*P* = 0.454), nor was there a MA by group interaction (*P* = 0.921) (Table 10, Figure 5C).

**Table 10:** **Peak grip force and mental age (MA)**.

**Effect**	**Estimate**	**Standard error**	**DF**	***t*-value**	***p***
**MAIN EFFECTS**					
MA					0.218
Group					0.454
Interaction					0.921
***POST-HOC***					
**Group**					
TD vs. ASD	0.43	0.373	124.00	1.16	0.247
DD vs. ASD	−0.02	0.365	124.00	−0.05	0.962
DD vs. TD	−0.45	0.421	124.00	−1.07	0.285
**Interaction**					
MA effect: TD vs. ASD	−0.002	0.01	124.00	−0.23	0.82
MA effect: DD vs. ASD	0.001	0.01	124.00	0.16	0.872
effect: DD vs. TD	0.004	0.01	124.00	0.40	0.69

TD, Typical Development; ASD, Autism Spectrum Disorders; DD, Developmental Delay.

### Aim 3: the association between experimental motor coordination variables and functional fine motor skills

The VABS and MSEL fine motor age equivalents were not significantly associated with any of the experimental motor coordination variables: grip to load force onset latency, time to peak grip force, grip force at onset of load force, or peak grip force (Table 11).

**Table 11:** **Effect of fine motor age equivalents (FMAE) on motor coordination variables**.

**Effect**	***F*-value**	***p***
**GRIP TO LOAD FORCE ONSET LATENCY**		
**Main Effects**		
Group	0.73	0.485
VABS FMAE	0.13	0.716
MSEL FMAE	0.07	0.797
**Interactions**		
Group × VABS FMAE	0.93	0.4
Group × MSEL FMAE	0.18	0.839
**TIME TO PEAK GRIP FORCE**		
**Main Effects**		
Group	0.72	0.491
VABS FMAE	1.42	0.236
MSEL FMAE	0.27	0.601
**Interactions**		
Group × VABS FMAE	0.33	0.72
Group × MSEL FMAE	0.07	0.937
**GRIP FORCE AT ONSET OF LOAD FORCE**		
**Main Effects**		
Group	2.27	0.108
VABS FMAE	1.48	0.227
MSEL FMAE	0.42	0.521
**Interactions**		
Group × VABS FMAE	1.6	0.207
Group × MSEL FMAE	2.5	0.087
**PEAK GRIP FORCE**		
**Main Effects**		
Group	1.61	0.108
VABS FMAE	1.09	0.227
MSEL FMAE	0.06	0.521
**Interactions**		
Group × VABS FMAE	1.98	0.207
Group × MSEL FMAE	2.5	0.087

VABS, Vineland Adaptive Behavior Scale; MSEL, Mullen Scales of Early Learning.

## Discussion

This study adds to the growing literature documenting that children with ASD have difficulties with volitional movements involving simple grasp and reach-to-grasp sequences (Hughes, 1996; Mari et al., 2003). Furthermore, our study provides the first experimental evidence of motor coordination during precision grip in young children with ASD as compared to children with DD and those developing typically, and identifies maturational variables important for motor coordination. Cognitive maturation, as measured by MA in this study, appeared to be an important variable in predicting motor performance across groups, especially for grip to load force onset latencies (i.e., onset latencies between grip and load forces got shorter indicating better coordination with increasing mental abilities), and time to peak grip force, although the MA effects on time to peak grip force depended upon complex interactions between groups.

Our findings demonstrate that temporal coordination deficits involving prolonged grip to load force onset latencies and prolonged times to peak grip force (but not force deficits) are present in young children with ASD. Although we hypothesized that we would find deficits in timing and force, these two sets of variables may reflect different underlying neural mechanisms. Studies of “clumsy” children (e.g., Lundy-Ekman et al., 1991) provide some evidence that neural mechanisms are separable, such that timing deficits are more related to cerebellar functions, and force is more related to basal ganglia function. However, it is important to note that the temporal coordination deficits found in our study were not specific to ASD but are likely associated with generalized maturational delays also present in children with other DD. These results are consistent with literature in older populations of children with ASD indicating that greater motor deficits are noted at lower levels of intellectual functioning (e.g., Mari et al., 2003), but extend these findings to very young children with ASD and DD. Although ASD and DD groups could not be differentiated on their motor performance during the precision grip task, the neurophysiology underlying temporal dyscoordination during such fine motor volitional actions may or may not be similar between these groups. In addition, it is quite likely that various mechanisms (e.g., cortical maturation, neuromuscular functions, biochemical changes with age, etc.) contribute differentially to motor deficits at different stages of development, and across various clinical populations (e.g., Seidler et al., 2010). Thus, longitudinal studies are warranted to better understand the developmental trajectory of these fine motor volitional actions.

Clearly, deficits in the timing (e.g., time to peak grip force relative to object load) in the groups with ASD and DD cannot be explained by corresponding deficits in IQ alone. There may be other factors/variables in addition to MA which are involved in the development of the timing of peak grip force. One possible factor is a deficit in predictive/feed-forward control (Schmitz et al., 2003). The timing of maximal peak grip force is programmed utilizing previous experience about object load and requires incorporating this information in the motor program in an anticipatory or predictive, feed-forward manner for subsequent precision grip trials (Flanagan and Wing, 1993). In older children who are typically developing, the time to peak grip force is reduced and is indicative of better feed-forward control (David et al., 2009). In the current sample of young children with ASD and DD, the prolonged times to peak grip force are suggestive of a control mode that relies on reactive/feedback rather than predictive/feed-forward control especially given that this pattern is not improving with increasing MA. By contrast, the TD group shows development toward more adult-like patterns in time to peak grip force with increasing MA.

Surprisingly, we found no statistically significant associations between the fine motor functional skills measured by standardized assessments, and any of the four motor coordination variables assessed experimentally in this study. The items on the VABS and MSEL fine motor subscales include simple unilateral and bilateral hand manipulation of objects but do not necessarily provide fine-tuned data on quality of movement patterns beyond basic performance requirements. Many of these assessments measure fine motor functional performance on a binary scale, i.e., whether children can or cannot perform a task, or on a nominal scale of restricted range that reduces the variability of motor performance. Although the temporal and force coordination variables assessed in our experiment are fundamental to fine motor manipulations, they are scaled with much greater precision. Future studies should address this possibility and include more sensitive and contextually relevant measures of motor coordination that include speed, amplitude, accuracy, etc. that encompass the variability of motor performance. It is also possible that the association between the fine motor age equivalents and the motor coordination variables is non-linear, or that children learn alternate strategies to compensate for their motor coordination deficits when performing functional tasks.

The limitations of this study included a small DD group relative to the ASD and TD groups that may have affected power to detect group differences between ASD and DD, especially given that there were some trends towards significance in our data. Second, although we hypothesized group differences in force variables based on earlier studies demonstrating lower grip forces in ASD samples (e.g., Hardan et al., 2003) and our mean peak grip forces were somewhat lower in the ASD group relative to the TD group, findings did not reach significance. Likewise, neither CA nor MA had any significant effect on grip force at onset of load force, and the peak grip force. It may be that the amount of force required was well within the maximal grip force across children, and thus all children were able to apply appropriate forces and scale these forces with object lift-off and with varying object loads. Future studies could vary the forces more to increase sensitivity of this task. Likewise, the sensitivity of the standardized assessments may have been limited to detect subtle differences in timing or quality of functional fine motor skills, and thus more contextually relevant tasks are needed. Finally, this study was cross-sectional in nature and can only infer developmentally-related changes affecting motor coordination. Longitudinal studies are needed to further test developmental hypotheses regarding the origins and consequences of temporal dyscoordination in children with ASD and DD.

In conclusion, this is among the first studies to empirically quantify motor coordination deficits in young children with ASD compared to children with DD or TD using an experimental precision grip task. We document that young children with ASD present with some temporal coordination deficits during a grasping task that differentiate them from children with TD, but not necessarily from children with DD. Thus, these temporal coordination deficits are most likely due to generalized maturational deficits and are probably not unique to ASD. The current study cannot determine to what extent the underlying neurophysiology of temporal dyscoordination is similar or different between children with ASD and children with DD; thus future research needs to investigate the underlying neurophysiology and development of volitional fine motor grasping patterns examined in this study. Moreover, longitudinal studies would be helpful to further explore the development of precision grip in children with ASD compared to control groups, and test the extent to which non-linear changes or compensatory strategies are present and associated with development of functional fine motor skills as measured with standardized assessments.

### Conflict of interest statement

The authors declare that the research was conducted in the absence of any commercial or financial relationships that could be construed as a potential conflict of interest.
